# Anatomical Extent, Presentation, and Outcomes of Distant Metastases to the Infratemporal Fossa: A Systematic Review

**DOI:** 10.3390/cancers18203392

**Published:** 2026-10-20

**Authors:** Ahmed Rasim Bayramoglu, Ufuk Erginoglu, Umid Sulaimanov, Nafiye Sanlier, Irem Uslu, Can Hamzaoglu, Zeyad Kamar, Yerkebulan Serikkanov, Dilshod Muhammadvalievich Mamadaliev, Mokhira Jalolova, Maryam Sabah Al-Jebur, Ersin Ikizoglu, Abdullah Keles, Mustafa Kemal Baskaya

**Affiliations:** Department of Neurological Surgery, University of Wisconsin School of Medicine and Public Health, 600 Highland Avenue, Madison, WI 53792, USA; bayramogluahmedrasim@gmail.com (A.R.B.); erginoglu@wisc.edu (U.E.); nafiyesanlier@gmail.com (N.S.); iremuslu20046@gmail.com (I.U.); hamzaoglucan@outlook.com (C.H.); zeyad.kamar@std.ankaramedipol.edu.tr (Z.K.); serikkanov@wisc.edu (Y.S.); neurocerrah@gmail.com (D.M.M.); jalolova@wisc.edu (M.J.); aljebur@wisc.edu (M.S.A.-J.); ikizoglu@wisc.edu (E.I.); abdullah.keles@wisc.edu (A.K.)

**Keywords:** differential diagnosis, distant metastasis, infratemporal fossa, masticator space, skull base neoplasms, systematic review

## Abstract

**Background/Objectives:** Distant metastasis to the infratemporal fossa (ITF) is rare and can mimic primary skull-base tumors. We systematically reviewed reported distant ITF metastases. **Methods:** Following PRISMA 2020 (PROSPERO CRD420261422546), PubMed, Scopus, and Web of Science were searched through 12 June 2026, for reports of ITF metastasis from a distant primary tumor; direct extension from adjacent tumors was excluded. Data were summarized descriptively, and reports were appraised with the JBI Critical Appraisal Checklist for Case Reports. **Results:** Seventeen reports (17 patients) were included (median age 59 years). The lung was the most frequent primary site (*n* = 4), followed by the kidney, colorectum, and thyroid (*n* = 3 each); adenocarcinoma was the most common histology (*n* = 7). Facial pain (*n* = 9) and numbness (*n* = 6) predominated. Bone destruction was reported in 12 patients, and the maxillary sinus (*n* = 7), orbit (*n* = 6), pterygopalatine fossa (*n* = 5), middle cranial fossa (*n* = 5), and cavernous sinus (*n* = 4) were involved. The median reported interval to ITF metastasis was 36 months; 11 metastases were metachronous, and 11 patients had other metastatic disease during the reported course. Five patients underwent regional resection or debulking; radiotherapy was reported in eight, radioiodine in three, and systemic drug treatment in nine. At last follow-up, 11 patients were alive, four had died, and status was unknown in two. **Conclusions:** Distant ITF metastases may present with facial pain and cranial neuropathies. Prognosis is insufficiently characterized, and management should be individualized within a multidisciplinary team. The evidence is limited to case reports with incomplete reporting. No external funding was received.

## Simple Summary

The infratemporal fossa is a small space at the base of the skull, behind the cheek and jaw, through which several important nerves and blood vessels pass. Cancers that begin elsewhere in the body very rarely spread to this space, and when they do they can look exactly like a tumor that started there, so the wrong diagnosis is easily made. We systematically searched the published literature and identified 17 reported patients. We describe how these patients presented, which cancers the deposits had come from, how long after the original cancer they appeared, and how they were treated. Our aim is to help doctors recognize this rare situation earlier, so that patients receive the right treatment.

## 1. Introduction

The infratemporal fossa (ITF) is a region of complex skull base anatomy at the intersection of several surgical disciplines, including neurosurgery, otorhinolaryngology, and maxillofacial surgery. It is bounded superiorly by the greater wing of the sphenoid, anteriorly by the posterior wall of the maxillary sinus, laterally by the ramus of the mandible, medially by the lateral pterygoid plate, posteromedially by the parapharyngeal space, and inferiorly by the plane at which the medial pterygoid muscle attaches to the mandibular ramus [1,2]. Within these boundaries, it contains a dense convergence of critical neurovascular and muscular structures, including the maxillary artery and its branches, the pterygoid venous plexus, the mandibular division of the trigeminal nerve (V3), and the medial and lateral pterygoid muscles [1,2]. Its proximity to the internal carotid artery and internal jugular vein, together with the convergence of multiple critical neurovascular structures, renders pathology in this region potentially life-threatening and surgical intervention particularly challenging [3]. The ITF communicates with several adjacent compartments through discrete foramina and fissures, providing anatomical corridors for both tumor spread and surgical access [4].

The majority of ITF tumors arise from primary or locally invasive processes [5]. In a systematic review of 106 patients with ITF tumors, schwannomas (26%) and meningiomas (14%) were the most common pathologies; malignant tumors predominantly arose from adjacent structures—including the parotid gland, paranasal sinuses, and masticator space—through direct contiguous extension [6]. A separate retrospective cohort of 62 patients similarly demonstrated a predominance of benign histology, accounting for 74% of cases, with juvenile nasopharyngeal angiofibroma, adenoid cystic carcinoma, and schwannoma as the most frequent tumor types [7]. Distant metastases to the ITF represent a markedly distinct and exceedingly rare subset of this already uncommon pathology [5,8]. The deep, concealed location of the ITF and the nonspecific nature of its presenting symptoms—most commonly facial hypoesthesia, preauricular pain, and trismus—frequently lead to diagnostic delays, with early manifestations often misattributed to temporomandibular joint dysfunction or dental disease [5,6]. This diagnostic challenge is further compounded by the tendency of ITF metastases to closely mimic benign primary tumors on neuroimaging. Dural-based metastatic lesions may be radiologically indistinguishable from meningiomas, while soft tissue deposits can resemble schwannomas, often requiring tissue sampling to distinguish them [7,9]. Distant ITF metastases may occur in the setting of advanced systemic disease, but ITF-specific prognosis and comparative treatment effects remain insufficiently characterized [6,8]. Metastases from distant primaries have been studied in more detail at neighboring head and neck sites. A systematic review of 696 metastases to the oral and maxillofacial region found the mandible to be the most common site (44.9%), with breast and lung as the most frequent primaries in women and men, respectively, and a mean survival of 9.8 months [10]. A systematic review of 873 orbital metastases identified breast, melanoma, and prostate as the leading primaries and proptosis as the main symptom, with a median overall survival of 6 months [11]. Renal cell carcinoma alone has accounted for 250 reported metastases to the oral soft tissues [12]. The ITF lies between these regions and communicates with both the orbit and the maxillary sinus, but metastases to the ITF have not been reviewed separately.

Published reports of distant ITF metastases remain limited in number and describe several primary sites [13,14,15,16,17]. A 2022 systematic review of 106 ITF tumors did not analyze distant metastases separately [6]. To our knowledge, no PRISMA-compliant systematic review focused exclusively on distant metastases to the ITF has been published to date. We therefore aimed to synthesize patient-level anatomical extent, presentation, diagnostic confirmation, management, and outcomes of distant ITF metastases, separately from primary and locally invasive ITF tumors.

## 2. Materials and Methods

This study was conducted in accordance with the PRISMA (Preferred Reporting Items for Systematic Reviews and Meta-Analyses) 2020 statement on systematic reviews and meta-analyses [18] and registered in the PROSPERO database CRD420261422546. The protocol is available in the PROSPERO record, and no amendments were made to it after registration. Institutional review board approval was not required, as the study was based exclusively on previously published data; the study was conducted in accordance with the Declaration of Helsinki.

### 2.1. Search Strategy

A systematic literature search was performed in PubMed, Scopus, and Web of Science on 12 June 2026, without date restrictions. The search queried the original database-specific keyword combinations (e.g., “infratemporal fossa” and “masticator space”) in combination with terms for metastasis (e.g., “Neoplasm Metastasis” and “Metastasis”). Boolean operators (“AND” and “OR”) were used to maximize sensitivity and ensure comprehensive identification of relevant reports. The search query was adapted to the requirements of each database. The complete search strategy is provided in Table S1. No language restrictions were applied. In addition, the reference lists of all included reports were manually screened; no additional records were identified.

### 2.2. Eligibility Criteria

Reports describing patients with metastatic lesions originating from distant primary malignancies to the infratemporal fossa were eligible for inclusion. Review articles, editorials, commentaries, conference abstracts without full text, technical notes without patient-level data, cadaveric or animal studies, and retracted publications were excluded. Reports were included if they reported patients with metastatic disease involving the infratemporal fossa and provided at least one category of clinical, radiological, pathological, therapeutic, or outcome-related information.

Reports were excluded if they described primary infratemporal fossa and skull base neoplasms, direct contiguous extension into the infratemporal fossa from an adjacent primary malignancy (i.e., local invasion), regional lymph node metastases, tumor seeding rather than true metastatic spread, or lesions without histopathological or clinically established evidence of metastatic disease. Reports with insufficient patient-level information were also excluded. For reports describing a distant primary malignancy, involvement of the infratemporal fossa was classified as distant metastasis regardless of the proximate route of spread. This included hematogenous deposition or contiguous extension from an adjacent metastatic deposit, provided that the primary malignancy was anatomically distant and discontinuous from the regional lesion. The term “masticator space” was included in the search to broaden retrieval; it did not independently establish eligibility. Inclusion required anatomical ITF involvement documented by imaging, operative findings, or a sufficiently specific anatomical description in the source report. The ITF was defined as the space below the greater wing of the sphenoid, behind the posterior wall of the maxillary sinus, medial to the mandibular ramus, and lateral to the lateral pterygoid plate, containing the pterygoid muscles, the maxillary artery, and the mandibular nerve [1,2]. An anatomical description was accepted when it placed tumor in a named content of this space, such as the pterygoid muscles or pterygoid plates, rather than only in the masseter, temporalis, or mandibular ramus. One included report used the term “masticator space”; its lesion lay deep to the temporalis muscle and was inseparable from the pterygoid muscles. Where the source report allowed, each lesion was also classified as centered in the ITF or as extending into the ITF from an adjacent metastatic deposit; otherwise, it was recorded as not determinable. Lesions confined to neighboring compartments without a documented ITF component were excluded. Primary tumors of the head and neck (e.g., thyroid carcinoma) were eligible when the infratemporal fossa was affected by metastatic rather than locally invasive disease. When a metastatic case was reported within a mixed case series or technical note, only the individual patient-level metastatic case was extracted. Outcome availability alone was not an exclusion criterion.

### 2.3. Study Selection and Data Extraction

All retrieved records were imported into Rayyan (Rayyan Systems Inc., Cambridge, MA, USA) [19] for screening and duplicate removal. Two independent reviewers (I.U. and A.R.B.) performed title and abstract screening, and two other reviewers (Z.K. and U.S.) evaluated the full texts of the screened reports to establish final eligibility. Study selection adhered to PRISMA guidelines. Disagreements were resolved through discussion, and any unresolved conflicts were adjudicated by an additional reviewer (C.H.).

Data were extracted using a standardized Microsoft Excel spreadsheet (Microsoft Corporation, Redmond, WA, USA). Extracted variables included report characteristics, patient demographics, primary tumor type and histology, interval between primary diagnosis and infratemporal fossa metastasis, presenting symptoms, radiological findings, extent of local disease, intracranial extension, involvement of adjacent skull base structures, presence of additional metastatic disease, diagnostic approach, treatment modalities, symptom response, recurrence or progression, survival outcomes, and follow-up duration. Metastases identified at the time of, or within 6 months of, the reported primary disease episode were classified as synchronous; those identified thereafter were considered metachronous. When an occult primary was identified retrospectively in an earlier surgical specimen, the historical primary episode was used. Source-specific timing origins are stated in the Results (Section 3.3). Data were independently extracted by two reviewers (A.R.B. and I.U.). The authors of the included reports were not contacted for additional data. All three reviewers (A.R.B., I.U., C.H.) cross-checked the extracted dataset before analysis to ensure completeness and consistency. For clinical and radiological variables, documented positive findings were recorded. Each clinical and imaging variable was then coded as present, explicitly absent, or not reported.

### 2.4. Quality Assessment

Quality assessment was independently performed by two reviewers (C.H., I.U.) using the eight-item Joanna Briggs Institute (JBI) Critical Appraisal Checklist for Case Reports [20]. The two authors resolved any disagreements through discussion, and a third reviewer (Z.K.) settled persistent conflicts. The appraisal is presented as consensus judgments for each checklist item, without a numerical summary score, cut-off, or categorical overall grading. For Mancini et al. [8], appraisal concerned the individually described metastatic case extracted from a mixed surgical series, not the publication as a case series; no separate publication-level case-series appraisal was performed. Item-level judgments are provided in Table S2. These judgments address reporting completeness and do not establish the credibility of treatment-effect conclusions.

### 2.5. Statistical Analysis

All extracted data were compiled and analyzed using Microsoft Excel (Microsoft Corporation, Redmond, WA, USA). Descriptive statistics were used to summarize the data. Continuous variables were expressed as medians with interquartile ranges (IQRs), and categorical variables were reported as frequencies and percentages. Percentages use the full cohort of 17 patients unless an available-data denominator is explicitly stated. Missing continuous measurements were excluded from their summaries; unreported vital status was retained as a separate category. For clinical and radiological features, proportions describe documented positive findings using the full cohort of 17 patients. Unreported findings were not treated as negative; these frequencies indicate reporting of a feature, rather than its prevalence among systematically assessed patients. For each feature, the number of reports in which it was explicitly absent or not reported is also given. Given the limited number of cases and the heterogeneity of the included reports, no comparative or inferential statistical testing was performed. Vital status and follow-up were summarized descriptively from the individually reported follow-up durations of patients with available data; because time-to-event data suitable for Kaplan–Meier estimation were not consistently reported, no censoring-adjusted (time-to-event) analysis was performed. No sensitivity analysis was performed because the synthesis was descriptive. Reporting bias was not assessed formally because no meta-analysis was performed, and the certainty of the evidence was not graded because the evidence consisted only of case reports and no pooled effect estimates were generated. The completed PRISMA 2020 checklist is provided in Table S3.

## 3. Results

### 3.1. Study Selection

A total of 591 records were identified through database searching, including 129 from PubMed, 289 from Scopus, and 173 from Web of Science. Following removal of 201 duplicates, 390 records underwent title and abstract screening. Of these, 354 were excluded (19 ineligible publication types and 335 not meeting eligibility criteria). Thirty-six reports were sought for retrieval; the full texts of five could not be retrieved despite attempts through institutional access and library searches. The remaining 31 reports were assessed, and 14 reports were excluded because no eligible distant ITF metastatic case was reported (*n* = 12) or anatomical eligibility criteria were not met (*n* = 2). Outcome availability alone was not an exclusion criterion. Seventeen reports describing 17 individual patients were included, comprising case reports and one metastatic case extracted from a mixed surgical series (Figure 1).

### 3.2. Quality Assessment Results

Reporting completeness was assessed using the JBI Critical Appraisal Checklist for Case Reports [20]. The included reports [8,13,14,15,16,17,21,22,23,24,25,26,27,28,29,30,31] generally fulfilled most checklist items (Table S2). Adverse or unanticipated events (Q7) were not described in 13 reports, and the post-intervention clinical condition (Q6) was unclear in two; all other items were fulfilled in all reports. No report was excluded based on appraisal results. These assessments should not be interpreted as evidence that the reports can establish comparative treatment effects.

### 3.3. Report Characteristics

A total of 17 patients with infratemporal fossa metastases were identified from 17 reports. Reports originated from the USA, Italy, Greece, Taiwan, India, Malaysia, China, Japan, Spain, Pakistan, and Morocco and were published between 2002 and 2025. The median age at presentation was 59 years (IQR 49.0–67.0; range 34–82), and nine patients (52.9%) were male. Lung (*n* = 4, 23.5%) was the most common primary site, followed by kidney, colorectum, and thyroid (*n* = 3, 17.6% each). Adenocarcinoma was the most frequent histology (*n* = 7, 41.2%), followed by clear cell carcinoma (*n* = 3, 17.6%). Facial pain (*n* = 9, 52.9%) and facial numbness (*n* = 6, 35.3%) predominated. Patient, diagnostic, and anatomical characteristics are summarized in Table 1; management and outcomes are summarized in Table 2.

The median reported interval to ITF identification was 36 months (IQR 0.0–60.0; range 0–216). Source-specific time origins were retained: Coca Pelaz reported 216 months from thyroid surgery initially considered benign, with malignancy subsequently recognized through ITF pathology and review of the earlier specimen [27]; Stamates reported 18 months from treatment completion [29]. Eleven cases (64.7%) were classified as metachronous and six (35.3%) as synchronous using the reported primary disease history. For Coca Pelaz, the retrospectively identified primary tumor in the earlier thyroid specimen defined the historical primary episode. These intervals should not all be interpreted as elapsed time from a contemporaneously established cancer diagnosis. The reported intervals and follow-up are shown in Figure 2.

### 3.4. Clinical Presentation and Radiological Findings

Facial pain was the most common presenting symptom, reported in nine patients (52.9%), followed by facial numbness in six patients (35.3%). Trismus and proptosis/exophthalmos were each reported in three patients (17.6%), and headache in two patients (11.8%). Diplopia was documented in one patient (5.9%), whereas otologic symptoms (otalgia or hearing loss) were reported in two patients (11.8%).

Radiological findings demonstrated frequent bone and adjacent skull base involvement. Bone destruction was the most commonly reported feature, observed in 12 patients (70.6%). Maxillary sinus involvement was reported in seven patients (41.2%), while pterygopalatine fossa extension was reported in five patients (29.4%). Orbital involvement was documented in six patients (35.3%). Cavernous sinus involvement was reported in four patients (23.5%), middle cranial fossa involvement in five patients (29.4%), and pterygoid muscle involvement in three patients (17.6%). Bone destruction was explicitly absent in two patients and not reported in three. Each of the other anatomical sites was explicitly absent in no more than one report and was not reported in the remaining patients. Among the symptoms, facial pain was explicitly absent in two patients and not reported in six. The pattern of involvement in each patient is shown in Figure 3, and the present, explicitly absent, and not reported counts for each variable are given in Table S4.

### 3.5. Disease Burden and Metastatic Patterns

Metastatic disease at other sites before ITF identification was documented in four reports (23.5%); absence of a prior metastatic history was not established for every other patient. During the reported disease course, 11 patients (64.7%) had other reported metastatic or multifocal systemic tumor involvement, based on the clinical, radiological, and pathological descriptions in the source reports. Biopsy confirmation of every additional focus was not required. These findings were not uniformly present at initial ITF diagnosis: Coca Pelaz subsequently developed sphenoid and pituitary lesions [27]. Liu reported suspected atlas bone/nodal disease and a lung nodule considered a possible second primary [14]; Malis described multiple bilateral pulmonary carcinoid lesions and considered the lungs the probable origin [26]. These qualifications are retained in Table 2 rather than treating every site as an independently confirmed additional metastasis. Among the remaining six patients, Mancini had no additional disease detected on total-body CT [8], whereas five reports did not report additional distant disease; the latter category does not establish uniformly negative staging or confirmed isolated metastasis. Regional cervical N1b nodes in Morita were not counted as additional distant disease. Contiguous extension into adjacent compartments was not counted as another metastatic site. The lesion was centered in the ITF in seven patients [8,15,16,17,21,26,27] and extended into the ITF from an adjacent metastatic deposit in three: from the maxillary sinus in one [22] and from the orbit in two [23,28]. In the remaining seven, the source report did not allow this distinction (Table S5). These descriptions distinguish regional presentations without inferring an epicenter or route where the source does not establish one.

### 3.6. Diagnostic Approaches, Management Strategies, and Reported Outcomes

Regional diagnostic confirmation comprised histology in 14 patients (82.4%) and aspiration cytology in one (Agrawal [31], 5.9%); two regional lesions (Ekpendu [24] and Rafi [28], 11.8%) were not sampled. In Ekpendu, malignancy was established from lip and esophageal biopsies and the ITF focus was identified on PET/CT among disseminated lesions. In Rafi, the regional lesion was presumed metastatic in the setting of established chondrosarcoma. Both remain presumed ITF metastases rather than regionally tissue-confirmed lesions. Reported immunohistochemistry supported primary origin, including thyroid transcription factor 1 (TTF-1) in Chaudhuri [23], thyroglobulin/TTF-1 in Coca Pelaz [27], estrogen/progesterone receptors in Stamates [29], and paired box 8 (PAX8)/CD10 in Ye [30]. Primary-tumor concordance was assessed where reported, including comparison with the earlier rectal tumor in Hsu [25] and retrospective thyroid-specimen reassessment in Coca Pelaz [27]. Regional sampling is distinct from sampling the primary tumor or another metastatic site. Reported regional sampling included incisional biopsy in Liu [14], core (Tru-Cut) biopsy in Hsu [25], transoral biopsy of temporal/pterygoid-region tissue in Malis [26], and fine-needle aspiration in Agrawal [31]. The site sampled, the immunohistochemical markers, and any comparison with the primary tumor are given for each patient in Table S5.

Five patients (29.4%) underwent documented regional resection or debulking [8,15,27,29,30]. Primary thyroid surgery in Agrawal and diagnostic biopsy were excluded from this count; the absence of resection in every other case was not assumed. Table S6 summarizes surgical context, approaches, resection extent, and reported morbidity. After regional diagnosis, external-beam radiotherapy was reported in six patients, radiotherapy of unspecified modality in two, and radioiodine in three. One patient received radiosurgery for a subsequent pituitary metastasis. Systemic drug treatment was documented in nine patients: cytotoxic chemotherapy in four, targeted therapy in three, unspecified chemotherapy in one, and endocrine therapy in one. An additional report contained conflicting chemotherapy documentation. Historical treatment and plans or referrals without documented administration were excluded from these counts. Treatment categories overlap and treatment targets differ (Table 2 and Table 3).

At the time of last follow-up, 11 patients (64.7%) were reported alive, four patients (23.5%) had died, and survival status was unavailable for two patients (11.8%). Overall survival data were incompletely reported, and a reliable median overall survival could not be estimated because most patients were alive at last contact and time-to-event data were sparse. Among the four patients who died, the interval from infratemporal fossa diagnosis to death was documented in three (approximately 4, 4, and 6 months), whereas reported follow-up among survivors ranged from 3 to 72 months. These descriptive figures were not adjusted for differences in follow-up duration and should therefore be interpreted with caution.

Regional clinical response or symptom status was described in 5/17 reports, and radiological response or stability in 4/17; these groups overlapped. Morita additionally reported no evidence of disease at 12 months. Case-specific outcomes and assessment anchors are summarized in Table 2, including pain or symptom change, lesion-volume response, and local recurrence or progression where reported. Objective cranial nerve recovery was not separately documented. These are information-availability counts, not favorable-response rates, and the heterogeneous observations do not support pooled treatment-effect estimates.

## 4. Discussion

This review, the first systematic review focused exclusively on this entity to our knowledge, identifies distant ITF metastasis as a rare, clinically distinct subgroup. Among 17 patients, lung was the leading primary site, followed by kidney, colorectum, and thyroid; facial pain and numbness were the dominant symptoms, and bone destruction was the most frequent imaging finding. Most lesions were metachronous, and 11 patients had other reported metastatic or multifocal systemic tumor involvement during the reported disease course. These observations support diagnostic vigilance and individualized multidisciplinary care, but heterogeneous and incomplete follow-up precludes firm prognostic or comparative treatment conclusions.

The findings differ from the general ITF literature. In the review by Bin-Alamer et al. benign schwannomas and meningiomas predominated, and malignant disease usually arose by contiguous extension [6]. In contrast, the present cohort comprised distant malignancies, usually after a latent interval and often with disseminated disease. Distant metastases should therefore be considered a separate biological and clinical category rather than a variant of the primary or locally invasive tumors that dominate general ITF series.

### 4.1. Diagnostic Challenges and Radiological Mimicry

Metastatic skull-base deposits may masquerade as benign primary tumors clinically and radiologically. In intracranial dural components, well-defined, homogeneously enhancing masses or a dural tail may resemble meningioma [32,33]. Predominantly extracranial ITF lesions may also resemble other primary tumors; the intracranial dural-lesion literature should not be generalized to all ITF deposits [6,7].

Lytic bone destruction may raise suspicion for metastasis in the differential diagnosis of a dural-based mass [32,33,34]. The cited vasogenic edema, adjacent leptomeningeal enhancement, and contrast-enhanced fluid-attenuated inversion recovery (FLAIR) rim findings concern intracranial dural lesions [34,35,36]. These specific signs were not documented in the extracted cases, and their diagnostic value for predominantly extracranial ITF metastases remains unestablished.

The present cohort is consistent with this emphasis on osseous behavior: bone destruction was the single most frequently reported radiological feature, documented in 12 of 17 patients (70.6%), and was in most instances accompanied by extension into an adjacent compartment (Figure 3) rather than by the expansile remodeling characteristic of a slow-growing benign lesion. Conversely, bone destruction was not documented in five patients, and its absence on imaging should not be taken as reassurance.

No single imaging feature is fully specific; imaging cannot reliably exclude metastatic disease [34]. Postoperative granulomas may mimic recurrence in previously operated intracranial lesions, and positron emission tomography/single-photon emission tomography (PET/SPET) may complement conventional neuroradiology in this setting [37]. This postoperative intracranial evidence does not establish ITF-specific diagnostic accuracy or validate unsampled ITF lesions.

The discriminative value of osseous change is also context dependent. In a consecutive series of 62 tumors arising within the ITF, bone erosion did not separate benign from malignant lesions, and neural invasion on MRI was the only imaging sign associated with malignancy [7]. Lytic destruction is therefore most useful when the question is whether a dural-based mass is a metastasis rather than a meningioma, and less useful as a general marker of malignancy among lesions of the fossa.

Mechanistically, when a dural tail accompanies a metastatic skull base deposit, it usually reflects a reactive dural change induced by adjacent tumor rather than a true meningothelial proliferation, which helps to explain why such lesions can convincingly mimic meningioma on imaging [32,38,39].

### 4.2. The Indispensable Role of Histopathology

The deep location and nonspecific symptoms of ITF lesions can delay imaging, and an indolent presentation should not be assumed to indicate benign biology [5,9]. Histopathology with a context-appropriate immunohistochemical panel remains central [9]. Diagnostic uncertainty may persist even after resection, and discordance between the clinical course and the original skull-base diagnosis should trigger pathological reassessment, even in patients without a known primary malignancy.

Tissue diagnosis also carries a second burden in this setting, because the specimen must establish not only malignancy but also the site of origin. In a deposit discovered before the primary tumor is known, morphology alone is rarely sufficient, and the immunophenotype directs the subsequent systemic work-up. This consideration is not academic in the present cohort, in which four primary sites accounted for 13 of the 17 patients and in which five reports recorded a zero-month interval to ITF identification. In this review, regional tissue confirmation was available in 15 of 17 patients (14 histological and one cytological), whereas two regional lesions remained presumed metastases based on the clinical and imaging context. Immunohistochemistry and primary-tumor concordance, where reported, supported origin assignment (Section 3.6).

### 4.3. Clinical Presentation and Anatomical Correlation

Trigeminal symptoms predominated, with facial pain (52.9%) and numbness (35.3%) most frequent. This is consistent with the relationship of the ITF to V2 and V3, which traverse the pterygopalatine and infratemporal corridors and enter the skull base through the foramina rotundum and ovale before converging at Meckel’s cave [40,41]. Compression, infiltration, or perineural spread may produce neuropathic pain and sensory loss, but symptoms alone do not distinguish these mechanisms [41]. Intraneural tumor was documented in Stamates [29]; this case-specific observation cannot be generalized to all patients.

These branches also provide routes for perineural spread, which may appear as nerve thickening or enhancement, foraminal enlargement, loss of fat planes, and denervation changes in the masticatory muscles [40,42,43].

Trismus and radiologically described pterygoid muscle involvement were each reported in three patients. Trismus may reflect masticatory muscle infiltration or reactive spasm, although a neural contribution cannot be excluded. Skeletal-muscle infiltration was histologically documented in Malis [26], but the mechanism was not directly established in every case. Limited mouth opening may be an early objective sign of a deep lesion in a patient whose pain has been attributed to a dental or temporomandibular cause [5,7].

### 4.4. Anatomical Corridors of Spread

The reported involvement can be interpreted in relation to the anatomical communications of the infratemporal fossa. The fossa is continuous anteriorly with the pterygopalatine fossa through the pterygomaxillary fissure, superiorly with the middle cranial fossa through the foramina ovale and spinosum, and with the orbit through the inferior orbital fissure, while the posterior wall of the maxillary sinus forms its anterior osseous boundary [1,2,4]. These relationships are reflected in the reported frequencies: after bone destruction, the maxillary sinus was the most frequently involved neighboring structure (seven patients, 41.2%), followed by the orbit (six patients, 35.3%), and the pterygopalatine and middle cranial fossae (five patients each, 29.4%), with cavernous sinus involvement in four (23.5%). These cross-sectional involvement patterns do not establish the direction or sequence of spread. Intracranial sites contribute to the total extent count, and extracranial extent has not been validated as a predictor of intracranial involvement.

### 4.5. Spectrum of Primary Tumors and the Biology of Metastatic Spread

Lung was the leading primary source, followed by kidney, colorectum, and thyroid. This distribution parallels larger series of head and neck metastases from infraclavicular primaries, in which lung, kidney, and breast predominate [44,45]. Renal cell carcinoma is particularly recognized for its disproportionate spread to the head and neck and skull base, a propensity attributed to its rich vascularity and hematogenous dissemination through valveless venous networks [46]. The distribution of primary sites also differed from that reported at neighboring sites. Breast cancer accounted for 36.3% of orbital metastases [11] and was the most frequent primary in women with oral and maxillofacial metastases [10], whereas no breast primary was found among the 17 patients in this review. Survival in those larger series was short, with a median of 6 months for orbital metastases [11] and a mean of 9.8 months for oral and maxillofacial metastases [10]; the incomplete survival data of the present review should be read against this background. Even when the ITF is involved by short-range extension from an adjacent deposit, a discontinuous relationship to a distant primary distinguishes metastatic disease from direct local invasion.

### 4.6. Management Strategies and Outcomes

Five patients underwent regional resection or debulking. Reported radiation modalities included external-beam radiotherapy (six patients), unspecified radiotherapy (two), radioiodine (three), and radiosurgery for a later pituitary lesion (one). Systemic drug treatment was documented in nine patients, with one additional report containing conflicting chemotherapy documentation. Treatment categories overlapped, targets differed, and plans or referrals were separated from documented administration (Table 2 and Table 3). Reported symptom and imaging responses were heterogeneous and incompletely assessed; treatment receipt alone does not demonstrate benefit or local control. Sparse follow-up precluded reliable survival or comparative treatment estimates.

Selected patients may benefit from focal treatment for symptom relief or local control, although the available reports cannot establish efficacy. Decisions should balance neurological risk, systemic disease burden, expected morbidity, available systemic options, and patient goals within a specialist multidisciplinary team [47].

Approach selection should consider tumor topography and treatment goals. The metastatic cases included a fronto-orbitozygomatic approach for diagnosis and decompression in Mancini [8], semicoronal exposure with zygomatic-arch translocation in Morita [15], repeated open and endoscopic procedures in Coca Pelaz [27], staged open and expanded endonasal debulking in Stamates [29], and partial endoscopic prelacrimal-recess resection in Ye [30] (Table S6). Potential neurological or vascular morbidity and achievable decompression must be weighed against systemic disease burden and patient goals. The cadaver comparison [48] and schwannoma technical report [49] provide general anatomical and technical guidance; the metastatic reports do not establish approach superiority or validated corridor-selection rules.

### 4.7. Strengths and Limitations

Strengths include the focused distant-metastatic population, searches across three databases, and independent screening and extraction. Patient-level timelines and anatomical mapping provide a structured descriptive comparison of heterogeneous reports.

This study has several limitations that warrant consideration. First, the analysis is based exclusively on published case reports and small case series, which are inherently susceptible to selection and publication bias; cases with favorable outcomes or unusual features are more likely to be reported, potentially distorting the apparent clinical spectrum and prognosis of this entity. Second, the retrospective nature of the source data and the heterogeneity of reporting precluded uniform extraction of key variables—follow-up duration, treatment details, and metastatic burden were inconsistently documented across reports—limiting the completeness of the pooled dataset. Third, given the small number of cases and the marked clinical and histological heterogeneity, only descriptive statistical analysis was feasible; no comparative or survival analyses could be reliably performed, and reported survival figures should be interpreted with caution, as they were not adjusted for censoring. Finally, although the search was limited to three major bibliographic databases (PubMed, Scopus, and Web of Science), their combination—supplemented by hand-searching of the reference lists of all included reports—provides broad and overlapping coverage of the relevant literature; nonetheless, the possibility that relevant cases, particularly those published in non-indexed journals, were missed cannot be excluded.

Furthermore, because the synthesis relies on the diagnoses recorded in the source reports, any misclassification of the lesion or its primary origin in an original publication—an appreciable risk given the well-documented tendency of infratemporal fossa metastases to mimic more common benign entities such as meningioma or schwannoma—would be propagated into the present dataset and could not be independently corrected.

Diagnostic uncertainty remains for the two unsampled regional lesions. Most clinical and imaging findings were not reported in many of the source reports, so the reported frequencies are likely to underestimate their true occurrence.

## 5. Conclusions

By separating distant metastatic involvement from primary and locally invasive ITF tumors, this review integrates patient-level anatomical extent, presentation, diagnostic evidence, and management. Distant ITF metastases are rare and can mimic primary skull-base tumors. Facial pain, cranial neuropathies, and destructive imaging features should prompt consideration of metastasis, especially in patients with a history of malignancy. Imaging cannot establish the diagnosis alone; regional tissue confirmation and reassessment of an atypical clinical course are important when feasible. Management should be individualized within a multidisciplinary team, and larger, consistently reported series are needed to define outcomes.

## Figures and Tables

**Figure 1:**
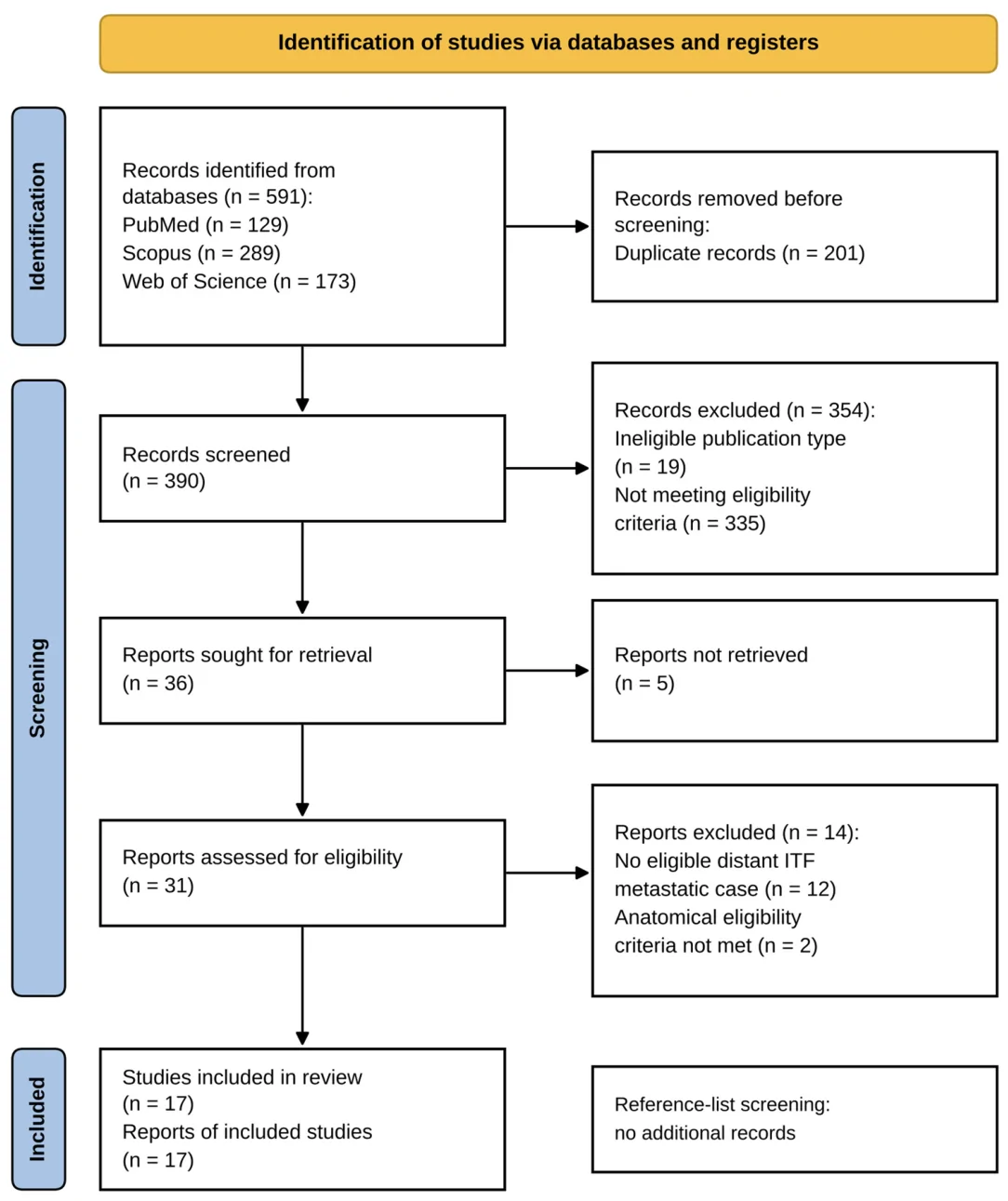
PRISMA 2020 flow diagram of report selection. ITF, infratemporal fossa. Database searching identified 591 records; after duplicate removal and screening, 31 reports were assessed and 17 were included. Fourteen reports were excluded because no eligible distant ITF metastatic case was reported (*n* = 12) or anatomical eligibility criteria were not met (*n* = 2). The full texts of five reports could not be retrieved despite institutional-access attempts and library searches. Reference-list screening identified no additional records; all 17 included reports were identified through database searching.

**Figure 2:**
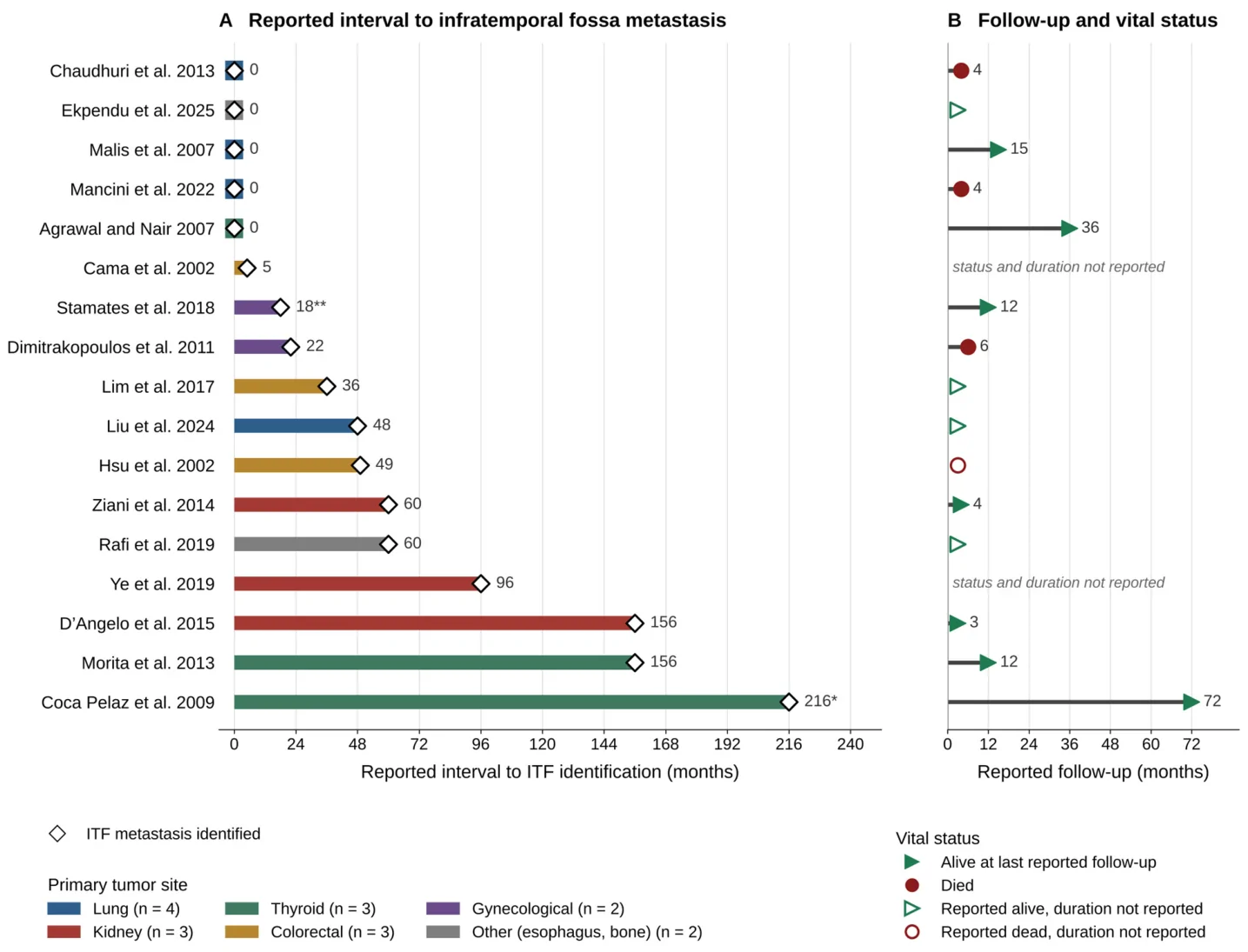
Reported intervals and follow-up for the 17 patients with distant ITF metastases. ITF, infratemporal fossa; NR, not reported. Patients are labeled by the first author and year of the source report [8,13,14,15,16,17,21,22,23,24,25,26,27,28,29,30,31]. (**A**) Reported interval to ITF identification, colored by primary site; open diamonds mark ITF identification. Five reports give a zero-month interval and Cama et al. [22] give five months. * Coca Pelaz et al. [27]: 216 months from thyroid surgery initially considered benign; the primary malignancy was recognized at ITF diagnosis after pathological reassessment. ** Stamates et al. [29]: 18 months from treatment completion. The median reported interval is 36 months (range 0–216); time origins are heterogeneous and are retained as reported. Coca Pelaz is classified as metachronous from the historical primary episode identified retrospectively in the earlier thyroid specimen. (**B**) Reported follow-up durations and last-contact vital status. Hollow markers indicate vital status reported without a follow-up duration. Duration was unavailable in seven cases, including Rafi [28], whose planned three-month review was not observed follow-up. Of ten reported durations, seven were at most 12 months. Heterogeneous assessment anchors and incomplete time-to-event data precluded survival estimation.

**Figure 3:**
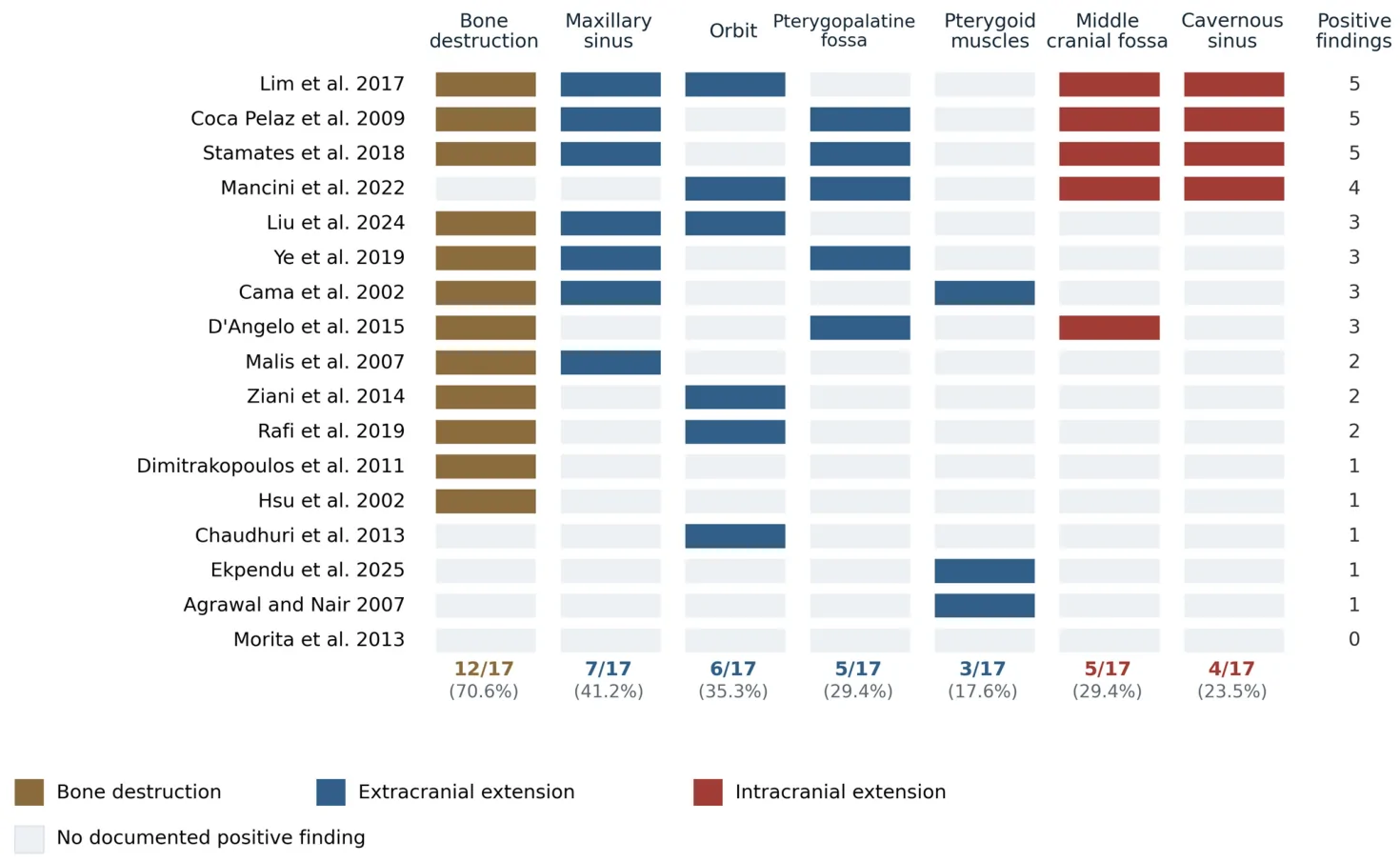
Documented anatomical involvement in the 17 patients with distant ITF metastases. ITF, infratemporal fossa; PPF, pterygopalatine fossa; MCF, middle cranial fossa; CS, cavernous sinus. Patients are labeled by the first author and year of the source report [8,13,14,15,16,17,21,22,23,24,25,26,27,28,29,30,31]. Each row represents a patient and each column a finding. Filled cells indicate a documented positive finding; pale cells indicate no documented positive finding and do not distinguish explicitly absent from unreported involvement. Row totals count documented findings, and percentages use all 17 patients as the denominator. Bone destruction remains documented in 12/17 (70.6%). Colors distinguish bone destruction, extracranial involvement, and intracranial involvement. Intracranial sites contribute to the row total; the heatmap does not establish the sequence of spread or validate extracranial extent as a predictor of intracranial disease.

**Table 1:** Patient, diagnostic, and anatomical characteristics of the 17 patients with distant ITF metastases.

Report	Age (y)	Sex	Presenting Symptoms	Primary Site	Histology	Imaging and Anatomical Findings
Ziani et al., 2014 [21]	67	Male	Facial pain + facial numbness + diplopia	Kidney	Clear cell carcinoma	Orbital involvement + bone destruction + infratemporal fossa
Cama et al., 2002 [22]	57	Female	Facial pain and otalgia	Sigmoid colon	Adenocarcinoma	Bone destruction + maxillary sinus invasion + involvement of the pterygoid muscles + infratemporal fossa
Chaudhuri et al., 2013 [23]	46	Female	Proptosis + cheek swelling	Lung	Adenocarcinoma	Orbital involvement + infratemporal fossa
D’Angelo et al., 2015 [17]	67	Male	Facial pain	Kidney	Clear cell carcinoma	Bone destruction + maxillary sinus remodeling + middle cranial fossa erosion + pterygopalatine fossa extension + infratemporal fossa
Dimitrakopoulos et al., 2011 [16]	37	Female	Facial pain + trismus	Uterine cervix	Squamous cell carcinoma	Bone destruction + infratemporal fossa
Ekpendu et al., 2025 [24]	82	Male	Regional symptoms NS	Esophagus	Adenocarcinoma	Pterygoid invasion + infratemporal fossa
Hsu et al., 2002 [25]	79	Female	Cheek mass	Rectum	Adenocarcinoma	Bone destruction + infratemporal fossa
Lim et al., 2017 [13]	49	Female	Headache + facial numbness	Rectum	Adenocarcinoma	Bone destruction + maxillary sinus invasion + orbital involvement + cavernous sinus involvement + middle cranial fossa involvement + infratemporal fossa
Liu et al., 2024 [14]	50	Male	Facial pain + facial numbness + trismus	Lung	Adenocarcinoma	Bone destruction + maxillary sinus invasion + orbital involvement + infratemporal fossa
Malis et al., 2007 [26]	43	Male	Trismus	Lung (probable primary)	Atypical carcinoid	Bone destruction + maxillary sinus invasion + infratemporal fossa
Mancini et al., 2022 [8]	59	Male	Facial pain (trigeminal neuralgia, V2/V3) + vision loss + proptosis	Lung	Adenocarcinoma	Orbital involvement + cavernous sinus involvement + middle cranial fossa involvement + pterygopalatine fossa extension + infratemporal fossa
Morita et al., 2013 [15]	74	Male	Regional symptoms NS	Thyroid	Papillary carcinoma	Infratemporal fossa
Coca Pelaz et al., 2009 [27]	61	Female	Facial pain + facial numbness + hearing symptoms	Thyroid	Follicular carcinoma	Bone destruction + maxillary sinus invasion + cavernous sinus involvement + middle cranial fossa involvement + pterygopalatine fossa extension + infratemporal fossa
Rafi et al., 2019 [28]	56	Male	Eye swelling or proptosis + facial pain + vision loss	Bone (toe)	Chondrosarcoma	Bone destruction + Orbital involvement + infratemporal fossa
Stamates et al., 2018 [29]	60	Female	Headache + facial pain + facial numbness	Endometrium	Endometrial carcinoma	Bone destruction + maxillary sinus invasion + cavernous sinus involvement + middle cranial fossa involvement + pterygopalatine fossa extension + infratemporal fossa
Ye et al., 2019 [30]	62	Male	Facial numbness	Kidney	Clear cell carcinoma	Bone destruction + maxillary sinus invasion + pterygopalatine fossa extension + infratemporal fossa
Agrawal and Nair 2007 [31]	34	Female	Facial swelling (left parotid region)	Thyroid	Poorly differentiated carcinoma (background of papillary carcinoma)	Pterygoid muscle involvement + infratemporal fossa

ITF, infratemporal fossa; NS, not specified; V2/V3, maxillary/mandibular divisions of the trigeminal nerve. Colon and rectal primaries are grouped as colorectal (*n* = 3) in the text. Maxillary sinus remodeling in D’Angelo et al. [17] was not counted as invasion. Anatomical and symptom entries describe reported findings; missing information does not indicate a negative assessment.

**Table 2:** Management, systemic disease status, and reported outcomes in the same 17 patients and order as Table 1.

Report	Reported Interval to ITF Identification	Prior Primary Treatment	Regional or Metastatic Treatment	Other Reported Metastatic or Multifocal Disease	Patient Status and Reported Outcomes
Ziani et al., 2014 [21]	60 months	Radical nephrectomy	Targeted therapy: sunitinib	Lung and bone; additional disease documented at ITF diagnosis	Alive (4 months); regional response NS
Cama et al., 2002 [22]	5 months	Surgery + CTx	Post-regional administration NS; earlier palliative folinic acid/5-FU decision retained separately	Lymph nodes and liver; additional disease documented at ITF diagnosis	Status/follow-up and regional outcome NS
Chaudhuri et al., 2013 [23]	0 months	None	EBRT + cytotoxic chemotherapy	No additional distant disease reported at ITF diagnosis	Dead (approximately 4 months). Proptosis resolved 1 month after RT completion; lung-primary progression after 3 chemotherapy cycles, followed by death 5 weeks later.
D’Angelo et al., 2015 [17]	156 months	Nephrectomy	Targeted antiangiogenic therapy	No additional distant disease reported	Alive (3 months). Mild lesion-volume reduction at 3-month follow-up, insufficient for metastasectomy.
Dimitrakopoulos et al., 2011 [16]	22 months	Surgery + RT + CTx	Radiotherapy, modality NS + chemotherapy, regimen NS	Mediastinal lymph nodes; additional disease documented at ITF diagnosis	Dead (6 months); regional response NS
Ekpendu et al., 2025 [24]	0 months	None	Radiotherapy, modality NS + cytotoxic chemotherapy	Liver, bone, lungs, lymph nodes, muscles, gastrohepatic ligament; additional disease documented at ITF diagnosis	Alive (duration NS); general stability reported without an ITF-specific endpoint
Hsu et al., 2002 [25]	49 months	Surgery	EBRT; course incomplete	Bone, lung, liver; additional disease documented at ITF diagnosis	Dead (duration NS); regional response NS
Lim et al., 2017 [13]	36 months	Surgery + CTx + Targeted therapy	EBRT: WBRT. Systemic regimen planned; delivery unclear	Lung; additional disease documented at ITF diagnosis	Alive (duration NS). Marked headache relief after WBRT; assessment interval NS.
Liu et al., 2024 [14]	48 months	Surgery + Targeted therapy	Targeted therapy: third-generation EGFR-TKI (agent NS)	Clinically/radiologically suspected atlas bone and nodal disease; lung nodule considered possible second primary	Alive; asymptomatic 1 year after first facial-pain onset. Post-treatment assessment duration NS.
Malis et al., 2007 [26]	0 months	None	EBRT + cytotoxic chemotherapy	Multiple bilateral pulmonary carcinoid lesions; probable lung origin; individual primary/metastatic pulmonary sites not distinguished	Alive (15 months). No significant clinical improvement after RT; clinical/radiological stability 15 months after presentation.
Mancini et al., 2022 [8]	0 months	None	Regional resection/decompression. Chemotherapy documentation conflicts: prose reports 1 dose; source table states no chemotherapy	None detected on total-body CT	Dead (4 months); regional response NS
Morita et al., 2013 [15]	156 months	Surgery	Regional extirpation + radioiodine	Regional cervical lymph nodes (N1b); no additional distant disease reported	Alive (12 months); no evidence of disease, assessment method NS
Coca Pelaz et al., 2009 [27]	216 months from prior thyroid surgery initially considered benign; malignancy recognized at ITF diagnosis	Surgery	Regional resection and repeat operations + radioiodine. Later pituitary radiosurgery; TSH suppression separately reported	No additional distant disease reported initially; later sphenoid/pituitary lesions	Alive (72 months). Earlier local recurrences; later MRI showed no lesions (MRI interval NS); lesion-free status 19 months after later treatment.
Rafi et al., 2019 [28]	60 months	Amputation	EBRT + cytotoxic chemotherapy	Lungs, pleura, lymph nodes; additional disease documented at ITF diagnosis	Alive (duration NS). Planned 3-month review is not observed follow-up; regional response NS.
Stamates et al., 2018 [29]	18 months from treatment completion	Surgery + RT + CTx	Regional staged debulking + EBRT + endocrine therapy (tamoxifen, megestrol)	No additional distant disease reported	Alive and well (12 months after surgery). Residual volume continued to decrease on reported 3-month MRI.
Ye et al., 2019 [30]	96 months	Surgery	Regional partial resection after embolization. Referred for oncological treatment; administration NS	No additional distant disease reported	Status/follow-up and regional response NS
Agrawal and Nair 2007 [31]	0 months	None	Radioiodine; thyroidectomy/neck dissection were primary-site surgery, not ITF resection	Bone (rib), cervical nodes; distant bone metastasis documented	Alive (36 months). Regional swelling reduced after radioiodine (interval NS); doing well at 3 years despite elevated thyroglobulin.

ITF, infratemporal fossa; CTx, chemotherapy; EBRT, external-beam radiotherapy; RT, radiotherapy; WBRT, whole-brain radiotherapy; EGFR-TKI, epidermal growth factor receptor tyrosine kinase inhibitor; MRI, magnetic resonance imaging; CT, computed tomography; TSH, thyroid-stimulating hormone; NS, not specified; 5-FU, 5-fluorouracil. Categories overlap and count reported post-regional administration; prior treatment, plans/referrals, TSH suppression, and conflicting Mancini chemotherapy are excluded from aggregate drug counts. Radioiodine, EBRT, and later pituitary radiosurgery are separate modalities. No additional disease reported does not imply negative staging. Outcomes retain source-specific assessment origins; missing data do not establish absence or benefit.

**Table 3:** Management approaches reported for the 17 patients with distant ITF metastases.

Approach	Patients, *n* [References]	Reported Setting or Indication	Reported Outcome and Assessment Time
Regional resection or debulking	5 [8,15,27,29,30]	Diagnosis and decompression of refractory neuralgia; extirpation of an ITF-confined lesion; resection and repeat operations for recurrence; staged debulking of extensive symptomatic disease; partial resection after embolization (Table S6)	Residual volume decreasing on 3-month MRI and alive and well at 12 months (Stamates); no evidence of disease at 12 months (Morita); local recurrences, later lesion-free (Coca Pelaz); died at 4 months (Mancini); not reported (Ye)
External-beam radiotherapy	6 [13,23,25,26,28,29]	Regional irradiation; whole-brain radiotherapy in one patient (Lim)	Proptosis resolved 1 month after radiotherapy (Chaudhuri); marked headache relief, interval not reported (Lim); no significant clinical improvement, stable at 15 months (Malis); course incomplete (Hsu); not reported (Rafi)
Radiotherapy, modality not reported	2 [16,24]	Given together with chemotherapy	General stability without an ITF-specific endpoint (Ekpendu); not reported (Dimitrakopoulos)
Radioiodine	3 [15,27,31]	Differentiated thyroid carcinoma	Regional swelling reduced, interval not reported (Agrawal)
Radiosurgery	1 [27]	Later pituitary metastasis	Lesion-free 19 months after treatment (Coca Pelaz)
Cytotoxic chemotherapy	4 [23,24,26,28]	Lung, esophageal, and chondrosarcoma primaries	Progression of the lung primary after three cycles (Chaudhuri)
Targeted therapy	3 [14,17,21]	Renal cell carcinoma (sunitinib; antiangiogenic agent); lung adenocarcinoma (third-generation EGFR-TKI)	Mild reduction in lesion volume at 3 months (D’Angelo); asymptomatic, interval from treatment not reported (Liu); not reported (Ziani)
Chemotherapy, agent not reported	1 [16]	Cervical carcinoma	Not reported
Endocrine therapy	1 [29]	Endometrial carcinoma (tamoxifen, megestrol)	See regional resection (Stamates)

ITF, infratemporal fossa; MRI, magnetic resonance imaging; EGFR-TKI, epidermal growth factor receptor tyrosine kinase inhibitor. Categories follow Table 2, and patients may appear in more than one row. Most patients received combined treatment, so an outcome cannot be attributed to a single approach. Outcomes are listed as reported, with the assessment time where given; they are not response rates.

## Data Availability

All data supporting the findings of this study are available within the article and its Supplementary Materials. The data extracted from the included reports are available from the corresponding author upon reasonable request.

## Supplementary Materials

The following supporting information can be downloaded at https://www.mdpi.com/article/10.3390/cancers18203392/s1: Table S1: Search strategy; Table S2: JBI critical appraisal of the 17 included reports; Table S3: PRISMA 2020 checklist; Table S4: Coding of clinical and imaging findings as present, explicitly absent, or not reported in the 17 included reports; Table S5: Basis of ITF documentation, anatomical pattern, regional sampling, and immunohistochemistry for each included patient; Table S6: Regional surgery for distant infratemporal fossa metastases: context, approach, resection extent, and reported morbidity. References [8,13,14,15,16,17,18,21,22,23,24,25,26,27,28,29,30,31] are cited in the Supplementary Materials.

## Abbreviations

The following abbreviations are used in this manuscript:

CT	Computed tomography
FLAIR	Fluid-attenuated inversion recovery
IQR	Interquartile range
ITF	Infratemporal fossa
JBI	Joanna Briggs Institute
MRI	Magnetic resonance imaging
PAX8	Paired box 8
PET	Positron emission tomography
PRISMA	Preferred Reporting Items for Systematic Reviews and Meta-Analyses
PROSPERO	International Prospective Register of Systematic Reviews
SPET	Single-photon emission tomography
TTF-1	Thyroid transcription factor 1
V2/V3	Maxillary and mandibular divisions of the trigeminal nerve
