# Supplementary material for: Anatomical Extent, Presentation, and Outcomes of Distant Metastases to the Infratemporal Fossa: A Systematic Review
Source: Cancers (Basel). 2026 Oct 20;18(20):3392. doi: 10.3390/cancers18203392 (PMC13702343; doi:10.3390/cancers18203392)
Supplement: Supplementary file 1 [file cancers-18-03392-s001.zip › cancers-4603557-supplementary.pdf]

Supplementary Materials

Table S1. Search strategy

Searches were run in PubMed, Scopus, and Web of Science on June 12, 2026, without date or language restrictions. Set #3 is the query used for each database. Scopus was searched in titles, abstracts, and keywords (TITLE-ABS-KEY) and Web of Science in Topic (TS). The PubMed query was entered without field tags and therefore searched all fields; in PubMed, a quoted phrase that matches a Medical Subject Heading (MeSH), such as "Infratemporal Fossa" or "Neoplasm Metastasis", also retrieves records indexed under that heading, without explosion to narrower headings.

Database	n	Set	Query
PubMed	129	#1	`("Infratemporal Fossa" OR "Masticator Space")`
		#2	`("Neoplasm Metastasis" OR "Extranodal Extension" OR "Neoplasm Micrometastasis" OR "Neoplasm Seeding" OR "Neoplasms, Unknown Primary" OR "Neoplastic Cells, Circulating" OR "Metastase" OR "Metastases, Neoplasm" OR "Metastasis" OR "Metastasis, Neoplasm" OR "Neoplasm Metastases")`
		#3	`#1 AND #2`
Scopus	289	#1	`TITLE-ABS-KEY ("Infratemporal Fossa" OR "Masticator Space")`
		#2	`TITLE-ABS-KEY ("Neoplasm Metastasis" OR "Extranodal Extension" OR "Neoplasm Micrometastasis" OR "Neoplasm Seeding" OR "Neoplasms, Unknown Primary" OR "Neoplastic Cells, Circulating" OR "Metastase" OR "Metastases, Neoplasm" OR "Metastasis" OR "Metastasis, Neoplasm" OR "Neoplasm Metastases")`
		#3	`#1 AND #2`
Web of Science	173	#1	`TS = ("Infratemporal Fossa" OR "Masticator Space")`
		#2	`TS = ("Neoplasm Metastasis" OR "Extranodal Extension" OR "Neoplasm Micrometastasis" OR "Neoplasm Seeding" OR "Neoplasms, Unknown Primary" OR "Neoplastic Cells, Circulating" OR "Metastase" OR "Metastases, Neoplasm" OR "Metastasis" OR "Metastasis, Neoplasm" OR "Neoplasm Metastases")`
		#3	`#1 AND #2`

Table S2. JBI critical appraisal of the 17 included reports

Report	Q1	Q2	Q3	Q4	Q5	Q6	Q7	Q8
Agrawal et al. (2007)[31]	Yes	Yes	Yes	Yes	Yes	Yes	No	Yes
Cama et al. (2002)[22]	Yes	Yes	Yes	Yes	Yes	Unclear	No	Yes
Chaudhuri et al. (2013)[23]	Yes	Yes	Yes	Yes	Yes	Yes	No	Yes
Coca Pelaz et al. (2009)[27]	Yes	Yes	Yes	Yes	Yes	Yes	Yes	Yes

Report	Q1	Q2	Q3	Q4	Q5	Q6	Q7	Q8
D'Angelo et al. (2015)[17]	Yes	Yes	Yes	Yes	Yes	Yes	No	Yes
Dimitrakopoulos et al. (2011)[16]	Yes	Yes	Yes	Yes	Yes	Yes	No	Yes
Ekpendu et al. (2025)[24]	Yes	Yes	Yes	Yes	Yes	Yes	Yes	Yes
Hsu et al. (2002)[25]	Yes	Yes	Yes	Yes	Yes	Yes	No	Yes
Lim et al. (2017)[13]	Yes	Yes	Yes	Yes	Yes	Yes	Yes	Yes
Liu et al. (2024)[14]	Yes	Yes	Yes	Yes	Yes	Yes	No	Yes
Malis et al. (2007)[26]	Yes	Yes	Yes	Yes	Yes	Yes	No	Yes
Mancini et al. (2022)[8]	Yes	Yes	Yes	Yes	Yes	Yes	No	Yes
Morita et al. (2013)[15]	Yes	Yes	Yes	Yes	Yes	Yes	No	Yes
Rafi et al. (2019)[28]	Yes	Yes	Yes	Yes	Yes	Yes	No	Yes
Stamates et al. (2018)[29]	Yes	Yes	Yes	Yes	Yes	Yes	No	Yes
Ye et al. (2019)[30]	Yes	Yes	Yes	Yes	Yes	Unclear	Yes	Yes
Ziani et al. (2014)[21]	Yes	Yes	Yes	Yes	Yes	Yes	No	Yes

Q1–Q8 correspond to the eight items of the Joanna Briggs Institute (JBI) Critical Appraisal Checklist for Case Reports, defined below. Reports are ordered alphabetically. The appraisal of Mancini et al. concerns the individually described metastatic case extracted from a mixed surgical series; it is not a publication-level case-series appraisal. The appraisal is presented as consensus judgments for each checklist item, without a numerical summary score, cut-off, or categorical overall grading. These judgments address reporting completeness and do not establish the credibility of treatment-effect conclusions.

JBI Critical Appraisal Checklist for Case Reports: item definitions

Q1. Were the patient's demographic characteristics clearly described?

Whether the report describes the patient's age, sex, medical history, diagnosis, prognosis, previous treatments, past and current diagnostic test results, and medications. The setting and context may also be described.

Q2. Was the patient's history clearly described and presented as a timeline?

Whether the report describes the medical, family, and psychosocial history of the patient, including relevant genetic information, together with relevant past interventions and their outcomes.

Q3. Was the current clinical condition of the patient on presentation clearly described?

Whether the presenting condition is described in detail, including symptoms, frequency, and severity, and whether differential diagnoses were considered.

Q4. Were diagnostic tests or assessment methods and the results clearly described?

Whether sufficient information is provided to understand how the patient was assessed, including a clear description of the diagnostic tests used and, where appropriate, supporting images.

Q5. Was the intervention(s) or treatment procedure(s) clearly described?

Whether the treatment protocol is described in sufficient detail for other clinicians to understand it, including drug type, route, dosage, frequency, and any side effects where relevant.

Q6. Was the post-intervention clinical condition clearly described?

Whether the clinical condition after treatment is described in terms of the presence or absence of symptoms, supported by images or figures where appropriate.

Q7. Were adverse events (harms) or unanticipated events identified and described?

Whether adverse and unanticipated events are documented and described, particularly when a new condition is treated or a new drug or technique is used.

Q8. Does the case report provide takeaway lessons?

Whether the report summarises the key lessons from the case, including background on the condition and practical guidance for clinicians facing similar cases.

Table S3. PRISMA 2020 checklist

Locations refer to the sections, tables, and figures of the main manuscript.

Section and topic	Item	Checklist item	Location where item is reported
**TITLE**			
Title	1	Identify the report as a systematic review.	Title
**ABSTRACT**			
Abstract	2	See the PRISMA 2020 for Abstracts checklist.	Abstract
**INTRODUCTION**			
Rationale	3	Describe the rationale for the review in the context of existing knowledge.	Section 1, paragraphs 1–2
Objectives	4	Provide an explicit statement of the objective(s) or question(s) the review addresses.	Section 1, last paragraph
**METHODS**			
Eligibility criteria	5	Specify the inclusion and exclusion criteria for the review and how studies were grouped for the syntheses.	Section 2.2
Information sources	6	Specify all databases, registers, websites, organisations, reference lists and other sources searched or consulted to identify studies. Specify the date when each source was last searched or consulted.	Section 2.1
Search strategy	7	Present the full search strategies for all databases, registers and websites, including any filters and limits used.	Section 2.1; Table S1
Selection process	8	Specify the methods used to decide whether a study met the inclusion criteria of the review, including how many reviewers screened each record and each report retrieved, whether they worked independently, and if applicable, details of automation tools used in the process.	Section 2.3, paragraph 1
Data collection process	9	Specify the methods used to collect data from reports, including how many reviewers collected data from each report, whether they worked independently, any processes for obtaining or confirming data from study investigators, and if applicable, details of automation tools used in the process.	Section 2.3, paragraph 2
Data items	10a	List and define all outcomes for which data were sought. Specify whether all results that were compatible with each outcome domain in each study were sought (e.g. for all measures, time points, analyses), and if not, the methods used to decide which results to collect.	Section 2.3, paragraph 2

Section and topic	Item	Checklist item	Location where item is reported
	10b	List and define all other variables for which data were sought (e.g. participant and intervention characteristics, funding sources). Describe any assumptions made about any missing or unclear information.	Section 2.3, paragraph 2; Section 2.5; Table S4
Study risk of bias assessment	11	Specify the methods used to assess risk of bias in the included studies, including details of the tool(s) used, how many reviewers assessed each study and whether they worked independently, and if applicable, details of automation tools used in the process.	Section 2.4
Effect measures	12	Specify for each outcome the effect measure(s) (e.g. risk ratio, mean difference) used in the synthesis or presentation of results.	Section 2.5 (descriptive measures only: medians with IQR, frequencies and percentages)
Synthesis methods	13a	Describe the processes used to decide which studies were eligible for each synthesis (e.g. tabulating the study intervention characteristics and comparing against the planned groups for each synthesis (item #5)).	Section 2.5
	13b	Describe any methods required to prepare the data for presentation or synthesis, such as handling of missing summary statistics, or data conversions.	Section 2.5 (handling of missing and unreported data)
	13c	Describe any methods used to tabulate or visually display results of individual studies and syntheses.	Section 2.5; Tables 1–3; Figures 2 and 3
	13d	Describe any methods used to synthesize results and provide a rationale for the choice(s). If meta-analysis was performed, describe the model(s), method(s) to identify the presence and extent of statistical heterogeneity, and software package(s) used.	Section 2.5 (descriptive synthesis; no meta-analysis)
	13e	Describe any methods used to explore possible causes of heterogeneity among study results (e.g. subgroup analysis, meta-regression).	Not applicable (no meta-analysis; Section 2.5)
	13f	Describe any sensitivity analyses conducted to assess robustness of the synthesized results.	Section 2.5, last paragraph (no sensitivity analysis; reason given)
Reporting bias assessment	14	Describe any methods used to assess risk of bias due to missing results in a synthesis (arising from reporting biases).	Section 2.5, last paragraph (not formally assessed; reason given)
Certainty assessment	15	Describe any methods used to assess certainty (or confidence) in the body of evidence for an outcome.	Section 2.5, last paragraph (not graded; reason given)
**RESULTS**			
Study selection	16a	Describe the results of the search and selection process, from the number of records identified in the search to the number of studies included in the review, ideally using a flow diagram.	Section 3.1; Figure 1

Section and topic	Item	Checklist item	Location where item is reported
	16b	Cite studies that might appear to meet the inclusion criteria, but which were excluded, and explain why they were excluded.	Section 3.1; Figure 1 (reasons for full-text exclusion)
Study characteristics	17	Cite each included study and present its characteristics.	Section 3.3; Tables 1 and 2; Table S5
Risk of bias in studies	18	Present assessments of risk of bias for each included study.	Section 3.2; Table S2
Results of individual studies	19	For all outcomes, present, for each study: (a) summary statistics for each group (where appropriate) and (b) an effect estimate and its precision (e.g. confidence/credible interval), ideally using structured tables or plots.	Tables 1–3; Figures 2 and 3; Tables S4–S6
Results of syntheses	20a	For each synthesis, briefly summarise the characteristics and risk of bias among contributing studies.	Sections 3.2 and 3.3
	20b	Present results of all statistical syntheses conducted. If meta-analysis was done, present for each the summary estimate and its precision (e.g. confidence/credible interval) and measures of statistical heterogeneity. If comparing groups, describe the direction of the effect.	Sections 3.3–3.6 (descriptive; no meta-analysis)
	20c	Present results of all investigations of possible causes of heterogeneity among study results.	Not applicable (no meta-analysis)
	20d	Present results of all sensitivity analyses conducted to assess the robustness of the synthesized results.	Not applicable (no sensitivity analysis; Section 2.5)
Reporting biases	21	Present assessments of risk of bias due to missing results (arising from reporting biases) for each synthesis assessed.	Not formally assessed (Section 2.5); publication bias discussed in Section 4.7
Certainty of evidence	22	Present assessments of certainty (or confidence) in the body of evidence for each outcome assessed.	Not graded (Section 2.5); limitations of the evidence in Section 4.7
**DISCUSSION**			
Discussion	23a	Provide a general interpretation of the results in the context of other evidence.	Section 4 (4.1–4.6)
	23b	Discuss any limitations of the evidence included in the review.	Section 4.7
	23c	Discuss any limitations of the review processes used.	Section 4.7
	23d	Discuss implications of the results for practice, policy, and future research.	Sections 4.6 and 5
**OTHER INFORMATION**			
Registration and protocol	24a	Provide registration information for the review, including register name and registration number, or state that the review was not registered.	Abstract; Section 2, paragraph 1 (PROSPERO CRD420261422546)

**Section and topic**	**Item**	**Checklist item**	**Location where item is reported**
	24b	Indicate where the review protocol can be accessed, or state that a protocol was not prepared.	Section 2, paragraph 1
	24c	Describe and explain any amendments to information provided at registration or in the protocol.	Section 2, paragraph 1
Support	25	Describe sources of financial or non-financial support for the review, and the role of the funders or sponsors in the review.	Funding statement
Competing interests	26	Declare any competing interests of review authors.	Conflicts of Interest statement
Availability of data, code and other materials	27	Report which of the following are publicly available and where they can be found: template data collection forms; data extracted from included studies; data used for all analyses; analytic code; any other materials used in the review.	Data Availability Statement; Tables S1, S2, S4, and S5

IQR, interquartile range. From: Page, M.J.; McKenzie, J.E.; Bossuyt, P.M.; et al. The PRISMA 2020 statement: an updated guideline for reporting systematic reviews. BMJ 2021, 372, n71. doi:10.1136/bmj.n71.

Table S4. Coding of clinical and imaging findings as present, explicitly absent, or not reported in the 17 included reports

Report	BD	MS	Orb	PPF	PtM	MCF	CS	FP	FN	Tr	Pr	HA	Oto	Dip
Agrawal and Nair (2007) [31]	NR	NR	NR	NR	P	NR	NR	NR	NR	NR	NR	NR	NR	NR
Cama et al. (2002) [22]	P	P	NR	NR	P	NR	NR	P	NR	NR	NR	NR	P	NR
Chaudhuri et al. (2013) [23]	NR	NR	P	NR	NR	NR	NR	A	A	A	P	A	A	A
Coca Pelaz et al. (2009) [27]	P	P	NR	P	NR	P	P	P	P	NR	A	A	P	A
D'Angelo et al. (2015) [17]	P	NR	NR	P	NR	P	NR	P	NR	NR	NR	NR	NR	NR
Dimitrakopoulos et al. (2011) [16]	P	NR	NR	NR	NR	NR	NR	P	NR	P	NR	NR	NR	NR
Ekpendu et al. (2025) [24]	NR	NR	NR	NR	P	NR	NR	NR	NR	NR	NR	NR	NR	NR
Hsu et al. (2002) [25]	P	NR	NR	NR	NR	NR	NR	NR	NR	NR	NR	NR	NR	NR
Lim et al. (2017) [13]	P	P	P	NR	NR	P	P	NR	P	NR	NR	P	NR	NR
Liu et al. (2024) [14]	P	P	P	NR	NR	NR	NR	P	P	P	NR	NR	NR	NR
Malis et al. (2007) [26]	P	P	NR	NR	NR	NR	NR	A	NR	P	NR	NR	NR	NR
Mancini et al. (2022) [8]	A	NR	P	P	NR	P	P	P	NR	NR	P	NR	NR	NR
Morita et al. (2013) [15]	A	A	NR	NR	NR	A	NR	NR	NR	NR	NR	NR	NR	NR
Rafi et al. (2019) [28]	P	NR	P	NR	NR	NR	NR	P	NR	NR	P	NR	NR	NR
Stamates et al. (2018) [29]	P	P	NR	P	NR	P	P	P	P	NR	NR	P	NR	NR
Ye et al. (2019) [30]	P	P	NR	P	NR	NR	NR	NR	P	NR	NR	NR	NR	NR
Ziani et al. (2014) [21]	P	NR	P	NR	NR	NR	NR	P	P	NR	NR	NR	NR	P
Present / absent / NR	12/2/3	7/1/9	6/0/11	5/0/12	3/0/14	5/1/11	4/0/13	9/2/6	6/1/10	3/1/13	3/1/13	2/2/13	2/1/14	1/2/14

P, present; A, explicitly reported as absent; NR, not reported. BD, bone destruction; MS, maxillary sinus; Orb, orbit; PPF, pterygopalatine fossa; PtM, pterygoid muscles; MCF, middle cranial fossa; CS, cavernous sinus; FP, facial pain; FN, facial numbness; Tr, trismus; Pr, proptosis; HA, headache; Oto, otologic symptoms (otalgia or hearing loss); Dip, diplopia. A finding was coded as present when the source report described it, as absent only when the report stated that it was absent or that the examination was otherwise normal, and as not reported in all other cases. Citations correspond to the main manuscript.

Table S5. Basis of ITF documentation, anatomical pattern, regional sampling, and immunohistochemistry for each included patient

Report	Basis of ITF documentation	Anatomical pattern	Regional sampling	Immunohistochemistry	Comparison with primary tumor
Agrawal and Nair (2007) [31]	CT and PET: masticator-space mass deep to the temporalis, inseparable from the pterygoid muscles	Not determinable	FNA of the parotid-region mass	NR	Cytology reported as thyroid carcinoma metastasis; no direct comparison
Cama et al. (2002) [22]	CT: mass involving the maxillary sinus, ITF, and pterygoid muscles	Extension from maxillary sinus deposit	Biopsy of the oral extension	CAM5.2, CEA, Ber-EP4, AE1/AE3, CK20 positive; CK7 negative	Morphology and immunophenotype matched the abdominal specimens
Chaudhuri et al. (2013) [23]	CT: orbital mass extending to the ITF	Extension from orbital deposit	Biopsy of the orbital mass	TTF-1 positive	Lung biopsy also adenocarcinoma; no direct comparison
Coca Pelaz et al. (2009) [27]	MRI; tumor occupying the ITF at operation	ITF-centered	Biopsy of the nasal extension; resection specimen	Thyroglobulin and TTF-1 positive	Review of the earlier thyroid specimen showed occult follicular carcinoma
D'Angelo et al. (2015) [17]	MRI and CT: mass centered in the ITF	ITF-centered	Transoral incisional biopsy after embolization	NR	Consistent with the known renal primary
Dimitrakopoulos et al. (2011) [16]	CT: ITF mass extending to the temporal region	ITF-centered	Open biopsy of the temporal extension (FNA nondiagnostic)	NR	No direct comparison
Ekpendu et al. (2025) [24]	PET/CT: uptake in the right ITF pterygoid region	Not determinable	ITF not sampled (lip and esophageal biopsies)	NR	Not applicable
Hsu et al. (2002) [25]	CT: mass in the parotid space and ITF	Not determinable	Core (Tru-Cut) biopsy of the cheek/ITF mass	NR	Histology similar to the rectal primary
Lim et al. (2017) [13]	CT: ITF mass; endoscopic transmaxillary biopsy	Not determinable	Endoscopic biopsy of the ITF lesion	NR	NR
Liu et al. (2024) [14]	CT, MRI, and PET-CT: soft-tissue mass in the ITF	Not determinable	Incisional biopsy at the temporal muscle attachment	TTF-1 and Napsin A positive; CDX-2, PAX8, and PSA negative	No direct comparison; lung origin from history
Malis et al. (2007) [26]	MRI: lesion filling the ITF; biopsy of the pterygoid region	ITF-centered	Transoral biopsies of the temporal and pterygoid region; transbronchial biopsies	NSE, pancytokeratin, CK7, CAM5.2, synaptophysin positive; TTF-1 and CK20 negative	Same histology in the ITF and lung biopsies
Mancini et al. (2022) [8]	MRI and CT: ITF mass extending into the PPF, middle cranial fossa, and orbit; operation	ITF-centered	Surgical specimen	NR	NR
Morita et al. (2013) [15]	CT and MRI: lesion in the ITF; extirpation	ITF-centered	FNA (nondiagnostic; washout thyroglobulin >8000 ng/mL), then excision	NR	NR
Rafi et al. (2019) [28]	CT: orbital deposit extending to the ITF	Extension from orbital deposit	Not sampled	NR	Not applicable
Stamates et al. (2018) [29]	MRI: middle fossa and ITF mass; operation	Not determinable (authors suspected a PPF center)	Surgical specimen	ER, PR, and pankeratin positive	Similar to the earlier uterine specimen

Report	Basis of ITF documentation	Anatomical pattern	Regional sampling	Immunohistochemistry	Comparison with primary tumor
Ye et al. (2019) [30]	CT: mass in the ITF, PPF, and parapharyngeal region; operation	Not determinable	Frozen section and resection specimen	PAX8, CD10, CK, EMA, vimentin positive; CK7 and CK20 negative	No direct comparison
Ziani et al. (2014) [21]	CT and MRI: lytic ITF tumor	ITF-centered	Biopsy of the ITF tumor	NR	NR

ITF, infratemporal fossa; PPF, pterygopalatine fossa; CT, computed tomography; MRI, magnetic resonance imaging; PET, positron emission tomography; FNA, fine-needle aspiration; NR, not reported. Anatomical pattern: ITF-centered, the source report placed the center or origin of the lesion in the ITF; extension, the source report described a metastatic deposit in a neighboring compartment extending into the ITF; not determinable, the source report did not allow this distinction. Citations correspond to the main manuscript.

Table S6. Regional surgery for distant infratemporal fossa metastases: context, approach, resection extent, and reported morbidity

Report	Clinical context and approach	Reported resection extent	Operative events and postoperative morbidity
Mancini et al. (2022) [8]	Refractory neuralgia; diagnosis and decompression. One-piece fronto-orbitozygomatic approach.	Adherent medial epidural residual toward cavernous sinus/clinoid described in operative account, although GTR stated elsewhere.	Case-specific complications not reported. Mixed-series enophthalmos cannot be assigned to this patient.
Morita et al. (2013) [15]	ITF-confined lesion. Semicoronal approach with temporary zygomatic-arch translocation and regional muscle/mandibular-process resection.	Extirpation described without standardized GTR/STR assessment.	Postoperative morbidity not separately reported.
Coca Pelaz et al. (2009) [27]	Initial subtemporal preauricular surgery; later recurrence operations included midfacial degloving and endoscopic procedures.	Initial complete resection; subsequent sellar resection limited by bleeding.	Initial recovery uneventful. Later combined operation followed by tracheotomy for failed extubation and laparotomy for intestinal obstruction.
Stamates et al. (2018) [29]	Extensive symptomatic disease. Staged orbitozygomatic/anterior petrosectomy and expanded endonasal surgery.	Debulking with intended residual around critical structures.	Intentional trigeminal-nerve transection at pons. Detailed postoperative cranial nerve examination not reported.
Ye et al. (2019) [30]	Large hypervascular lesion. Embolization followed by endoscopic prelacrimal-recess approach.	Partial resection limited by approximately 1000 mL blood loss despite hemostatic measures.	Blood loss was an operative event. Detailed postoperative neurological morbidity not separately reported.

ITF, infratemporal fossa; GTR, gross total resection; STR, subtotal resection. Citations correspond to the main manuscript. The table summarizes five patients with documented regional resection or debulking; primary-site surgery and diagnostic biopsy are excluded. Operative events and intentional neural sacrifice are distinguished from postoperative complications. Unreported morbidity does not indicate absence of morbidity. Later procedures are identified where reported and should not be attributed to the initial operation.
